# Tuberculin Dispensaries and the Public

**Published:** 1913-05-10

**Authors:** 


### TUBERCULIN DISPENSARIES AND THE PUBLIC.

We need not point out the fallacy of the arguments which have been used by many of those who prognosticate the eradication of consumption as the probable immediate result of the passing of this Act and the application of its sanatorium clauses. Medical men who have studied the matter know that it is not easy to eradicate a disease by Act of Parliament, and that success by such methods can only be hoped for if the special legislative measure has been drafted with exceeding great care and after the most conscientious consideration of the various lines of attack that may be planned. No one can honestly say that the hastily evolved National Insurance Act is an example of such conscientious legislation, and no one can honestly believe that with the date of its enforcement there dawns for the consumptive citizen an era of undreamed of betterment. Yet most of us, while fully recognising its deficiencies, will at the same time welcome its influence so far as this question of the treatment of consumption is concerned, inasmuch as it will undoubtedly cause the public to consider in some detail the various aspects of the great problem which so urgently awaits solution.

One of the recently much-advertised suggestions towards a solution has been the tuberculin dispensary. Here, again, propaganda work is largely responsible for the publicity which has been accorded to certain institutions which have identified themselves closely with pioneering work in this direction of treating consumptive patients by means of tuberculin. The adverse criticism which has been given of the methods has as yet impressed the medical man alone, because the expert aspects of the matter can only be fully comprehended and discussed by medical experts. The public at large imagines, and honestly imagines, that in the establishment of tuberculin dispensaries all over the country is to be found an almost ideal method for combating the disease. It does not as yet realise that such dispensaries can do very little to improve the condition of the advanced consumptive patient, and that their influence upon the incipient case is not always beneficial. Hitherto the profession has been chary of discussing the pros and cons of tuberculin treatment in a manner which can be followed and understood by the lay public; it has keenly and sharply criticised the methods of treatment in its own press and at its own meetings, but it has not taken the public into its confidence and shown clearly what are the dangers of slavishly following the methods of the dispensary. Yet many medical men can, if they like, give instances from their case-books showing how very real these dangers are. The question then arises whether it is right that the public should be permitted to continue in its confident belief that tuberculin dispensaries are harmless and highly laudable centres of treatment, whose place, were they once disestablished, cannot be taken by any other institution or centre of treatment. The advertisement of the dispensaries has been so insistent, and the agitation on their behalf backed by such powerful support, that no one can blame the laymen for holding such a belief.

While we do not question that some dispensaries have been the means of doing good work in many cases of tuberculosis, we are nevertheless convinced that many of them have also been the means of seriously curtailing the patients' chance of cure. We do not wish to dilate on the haphazard manner in which treatment is carried out at some dispensaries, upon the want of adequate accommodation for patients that exists at others, and upon the inconveniences entailed upon patients at yet a third class; these are matters which, once we grant that dispensary treatment is advisable and praiseworthy, may be left to the good sense of those who are responsible for the upkeep of these institutions. At present it is a well-known fact that some dispensaries are housed in rooms which are absolutely unsuitable for medical treatment of patients of any kind, and that the arrangements made for the comfort and convenience of those who attend them are primitive in the extreme.

